# Lithium chloride prevents interleukin‐1β induced cartilage degradation and loss of mechanical properties

**DOI:** 10.1002/jor.22913

**Published:** 2015-07-29

**Authors:** Clare L. Thompson, Habiba Yasmin, Anna Varone, Anna Wiles, C. Anthony Poole, Martin M. Knight

**Affiliations:** ^1^ Institute of Bioengineering and School of Engineering and Materials ScienceQueen Mary University of LondonLondonUnited Kingdom; ^2^ Dunedin School of MedicineUniversity of OtagoDunedinNew Zealand

**Keywords:** cartilage, mechanics, lithium chloride

## Abstract

Osteoarthritis is a chronic degenerative disease that affects the articular cartilage. Recent studies have demonstrated that lithium chloride exhibits significant efficacy as a chondroprotective agent, blocking cartilage degradation in response to inflammatory cytokines. However, conflicting literature suggests lithium may affect the physicochemical properties of articular cartilage and thus long‐term exposure may negatively affect the mechanical functionality of this tissue. This study aims to investigate the effect of lithium chloride on the biomechanical properties of healthy and interleukin‐1β treated cartilage in vitro and examines the consequences of long‐term exposure to lithium on cartilage health in vivo. Bovine cartilage explants were treated with lithium chloride for 12 days. Chondrocyte viability, matrix catabolism and the biomechanical properties of bovine cartilage explants were not significantly altered following treatment. Consistent with these findings, long term‐exposure (9 months) to dietary lithium did not induce osteoarthritis in rats, as determined by histological staining. Moreover, lithium chloride did not induce the expression of catabolic enzymes in human articular chondrocytes. In an inflammatory model of cartilage destruction, lithium chloride blocked interleukin‐1β signaling in the form of nitric oxide and prostaglandin E2 release and prevented matrix catabolism such that the loss of mechanical integrity observed with interleukin‐1β alone was inhibited. This study provides further support for lithium chloride as a novel compound for the treatment of osteoarthritis. © 2015 The Authors. *Journal of Orthopaedic Research* published by Wiley Periodicals, Inc. J Orthop Res 33:1552–1559, 2015.

Articular cartilage, the connective tissue within synovial joints, functions to reduce contact stresses to the underlying bone due to its ability to deform during normal loading and distribute applied forces. The biomechanical properties of cartilage, in particular its compressive moduli, are critical in providing the tissue with its mechanical functionality. The stiffness of the tissue is governed by the physicochemical interaction between collagen and hydrated proteoglycan which form the extracellular matrix (ECM). Disruption of the balance between ECM synthesis and catabolism occurs early in the pathogenesis of osteoarthritis (OA), a chronic degenerative disease affecting the joints, especially those with significant load bearing functions such as the hip and knee.^1^, ^2^ This causes a reduction in cartilage matrix stiffness. Ultimately, the softening of the tissue leads to surface fibrillations and osteoarthritic lesions which, in the presence of physiological loading, progress to complete tissue breakdown with associated joint pain and loss of mobility.^3^, ^4^ In the UK alone, one third of individuals over the age of 45 have sought treatment for OA which represents a significant burden for health services.^5^ However no therapeutic interventions currently exist that can prevent cartilage destruction.

Inflammation has been implicated in the early stages of OA development. The secretion of pro‐inflammatory cytokines such as interleukin‐1β (IL‐1β) and tumor necrosis factor α (TNFα) trigger a signaling cascade that stimulates the release of nitric oxide (NO) and prostaglandin E_2_ (PGE_2_). This leads to the production of proteolytic enzymes such as matrix metalloproteinase (MMP)‐13 and “a disintegrin and metalloproteinase with thrombospondin motifs” (ADAMTS)‐5 resulting in cartilage degradation.^6^ A recent in vitro study demonstrated that lithium chloride (LiCl), a compound commonly used for the treatment of bipolar disorder, exhibits chondroprotective properties in response to degradation induced by IL‐1β.^7^ In this study, Hui et al. demonstrate that LiCl selectively inhibits the phosphorylation and activation of p38 MAPK in response to IL‐1β thus preventing the induction of MMP‐1 and MMP‐13 and the resulting cartilage degradation that follows. More recently, Minashima et al. demonstrated that intra‐articular injection of LiCl into the joint cavity significantly reduces cartilage damage in mouse models following the surgical induction of OA.^8^ However, in other studies LiCl is shown to induce apoptosis^9^ and to disrupt the sulphation of glycosaminoglycans^10^ which is likely to impact on proteoglycan hydration and tissue mechanics. No published studies have examined the influence of LiCl on cartilage biomechanics which are so critical for tissue functionality. Furthermore, the long‐term effects of lithium treatment on joint health are unknown.

The present study examined the effect of long‐term dietary lithium on cartilage health in vivo using a rat model. In addition, the influence of LiCl on cartilage degradation and mechanical properties in healthy tissue and in the presence of the pro‐inflammatory cytokine IL‐1β was determined. This study demonstrates that LiCl does not significantly affect the mechanical properties of articular cartilage explants and that long‐term exposure to lithium does not adversely affect joint health nor induce arthritis in rats. Furthermore, in an inflammatory in vitro model of cartilage degradation, LiCl inhibits cartilage degradation and prevents the loss of mechanical integrity that occurs in response to IL‐1β. Thus, this study provides important new data suggesting that LiCl may have potential application as a novel treatment for preventing cartilage degradation and OA progression.

## MATERIALS AND METHODS

### Animal Studies

Male Wistar rats (three per treatment group) were used to examine the influence of dietary lithium on cartilage health. Tissue was obtained from rats fed a lithium enriched diet, 60 mmol lithium/kg food, for 9 months. Rats were initially given food containing 40 mmol lithium/kg dried food for the first 7 days, followed by 9 months at the higher dose enabling them to make physiological adjustments to the treatment regime. Age‐matched controls were kept on normal food and water. To minimize the lack of weight gain associated with lithium‐induced diuresis, rats were given continuous access to a salt block and free access to water. In order to closely monitor lithium serum levels, a blood sample was taken from the tail vein at the end of the experimental period. These studies were approved by the Otago University Animal Ethics Committee (AEC 98/10).

Rats were sacrificed by decapitation following a brief exposure to carbon dioxide. The left hind limb was detached and the musculature removed to expose the knee joint which was then fixed in 10% neutral buffered formalin for 48 h, decalcified in 10% EDTA and embedded in paraffin wax. To assess cartilage quality, 5 μm thick sections were cut perpendicular to the cartilage surface and stained with safranin O/fast green.

#### Isolation of Bovine Cartilage Explants and Chondrocyte Culture

**Figure 1:**
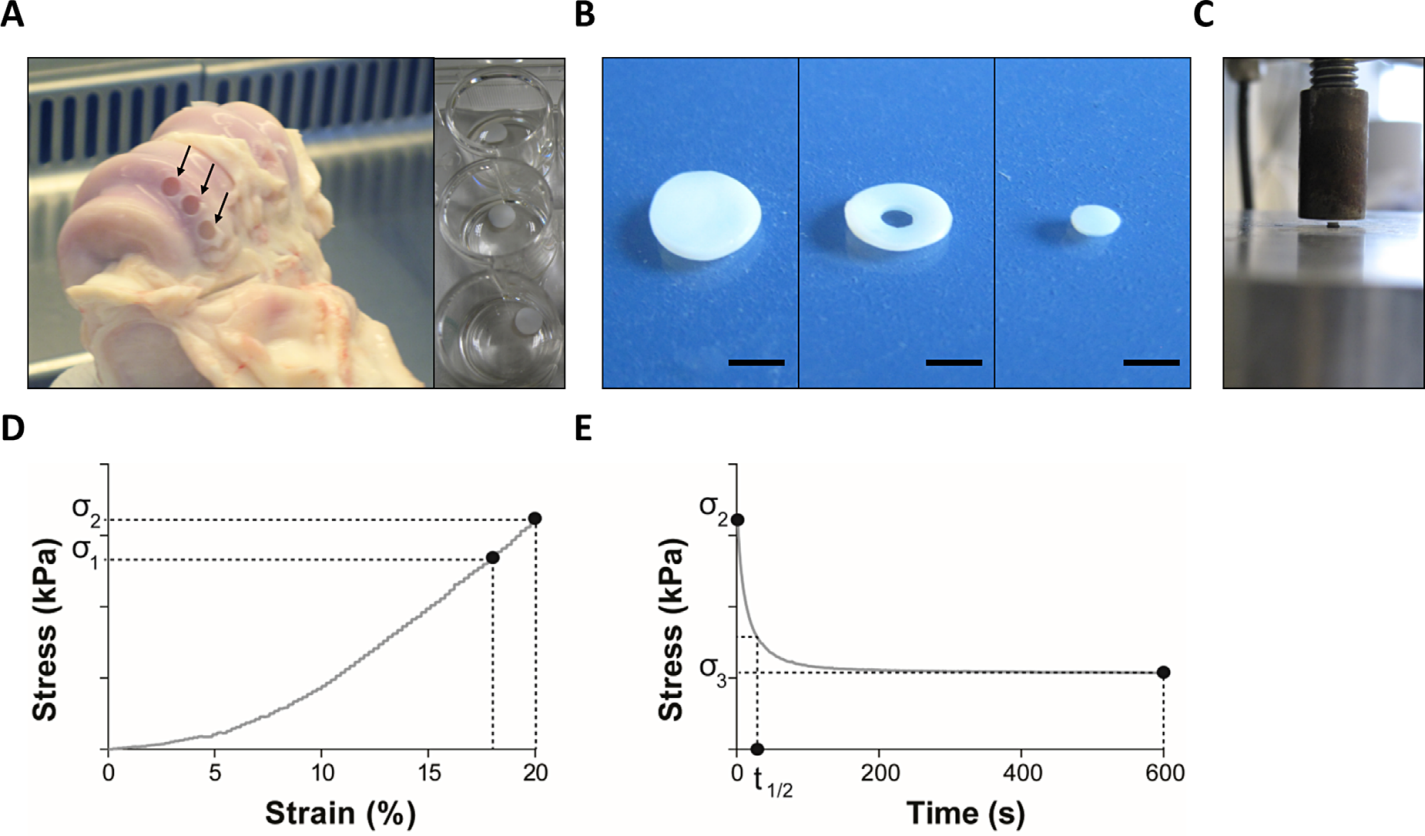
Cartilage explant model system. (A) Cartilage explants were removed from the proximal surface of the metacarpalphalangeal joint and cultured in 24‐well plates. Arrows indicate sites from which cylindrical explants were removed. For mechanical testing, (B) a 2 × 2 mm^2^ core was removed from the center of the explant to improve surface contact with the (C) 10 mm diameter testing platen. Scale bar = 2.5 μm. Representative (D) stress–strain and (E) stress–time plots showing the calculation of cartilage mechanical properties.

Forefeet from freshly slaughtered adult bovine steers (aged 18–24 months) were obtained from a local abattoir. Cartilage explants were isolated from the proximal surface of the metacarpal phalangeal joint (Fig. 1a) using a 5 × 5 mm^2^ biopsy punch. Explants were cultured in Dulbecco's Modified Eagles Medium (DMEM) supplemented with 10% (v/v) fetal calf serum (FCS), 1.9 mM l‐glutamine, 96 U/ml penicillin, 96 µg/ml streptomycin, 20 mM 2‐[4‐(2‐hydroxyethyl) piperazin‐1‐yl]ethanesulfonic acid (HEPES) buffer, and 0.74 mM l‐ascorbic acid (Sigma–Aldrich, Poole, UK), hereafter referred to as DMEM+10%FCS, at 37°C, 5% CO_2_. The explants were rested for 2 days then transferred to 24‐well plates (Fig. 1a) and cultured in 1 ml DMEM+10%FCS containing 0–50 mM LiCl (Sigma–Aldrich) for a further 12 days. Additional groups of cartilage explants at each LiCl concentration were cultured in 5 ng/ml IL‐1β (Peprotech, London, UK). The culture medium was collected every 3 days for biochemical analysis and replaced with fresh treatment medium.

Primary bovine articular chondrocytes were isolated by enzymatic digestion as previously described^11^ and cultured in DMEM+10%FCS at 37°C, 5% CO_2_ for 6 days without passage.

Primary human articular chondrocytes were purchased commercially (Articular Engineering, Northbrook, IL), cells were isolated from full depth cartilage slices removed from the femoral condyles of normal and osteoarthritic male donors aged 60–71 years old. Cells were cultured in chondrocyte culture medium (Articular Engineering) at 37°C, 5% CO_2_ and used in experiments from P0 to P3.

#### Determination of Chondrocyte Viability

Chondrocyte viability in cartilage explants was assessed at the end of the experimental period by live/dead staining. After 12 days, explants were incubated for 45 min at 37°C with 5 μM Ethidium homodimer‐1 (EthD‐1) to label dead cells and 5 μM Calcein AM, a marker for live cells. Explants were then washed in PBS and immediately imaged on a Leica SP2 confocal microscope.

### Quantitative Real‐Time PCR

Total RNA was isolated using an RNeasy Kit (Qiagen, Manchester, UK) and cDNA synthesis performed using the Quantitect reverse transcription kit (Qiagen) according to the manufacturer's protocol. For real‐time PCR, syber‐green PCR was performed using KAPA SYBR® FAST Universal 2X qPCR Master Mix (KAPA Biosystems, London, UK) according to the manufacturers protocol. An annealing temperature of 60°C was used for PCR reactions and fluorescence data were collected using the MX3000P QPCR instrument (Stratagene, Cheshire, UK). Data were analysed using the relative standard curve method^12^ and target genes were normalized to 18S ribosomal RNA. Primer sequences were as follows: ADAMTS‐5 For “CCTTGTGGAAAGGGGAGAAT,” ADAMTS‐5 REV “ACAGTGACGATAGGCAAACT,” MMP‐13 For “AGCCACTTTATGCTTCCTGA” MMP‐13 REV “TCAAACTGTATGGGTCCGTT,” 18S For “CGGCTACCACATCCAAGGAA,” and 18S REV “AGCTGGAATTACCGCGGC.”

#### Biochemical Analysis

Sulphated glycosaminoglycan (sGAG) release was assessed using the dimethylmethylene blue (DMMB) assay.^13^ The sGAG content for 40 μl samples of culture medium was assayed against a chondroitin sulphate (6‐sulphate:4‐sulphate; 0.33:1; Sigma–Aldrich) standard curve. Collagen release was assessed by determining the hydroxyproline content of the culture medium.^14^ The hydroxyproline content for 100 μl samples of culture medium was assessed against a hydroxyproline standard curve. A hydroxyproline‐to‐collagen ratio of 1:7.69 was used to calculate collagen content.^14^ The release of NO into the culture medium was monitored indirectly by measuring the levels of nitrite (NO_2_), a stable metabolite of NO, using the Griess assay as described previously.^15^ The nitrite content of 100 μl samples of culture medium was assayed against an NaNO_2_ standard curve.

#### Protein Isolation and Western Blotting

Protein isolation and western blotting were performed as described previously.^16^ The primary antibodies used in this study were: anti‐iNOS (1:200, Abcam: ab3523) and β‐Actin (1:5,000, Abcam: ab8226). Detection was performed using the Licor Odyssey Infra‐red imaging system.

#### ARGxx Neo‐Epitope Detection

Following isolation, cartilage explants were washed and rested for 2 days in serum‐free DMEM, following which they were transferred to 24‐well plates and cultured for a further 3 days in serum‐free DMEM containing 0–50 mM LiCl with or without IL‐1β (5 ng/ml). These conditions were identical to those used for biochemical analysis except for the absence of serum which was necessary for the detection of the ARGxx neo‐epitope. The medium was collected and deglycosylated overnight as previously described.^17^ Briefly, 150 μl medium was incubated with 0.01 U/ml chondroitinase ABC and 0.01 U/ml Keratanase (Sigma–Aldrich) in 50 μl deglycosylation buffer (50 mM Tris HCl, pH 7.5, 50 mM sodium acetate and 10 mM EDTA) at 37°C overnight. Following deglycosylation, the protein was lyophilized and subjected to SDS–PAGE and western blotting with anti ARGxx neo‐epitope antibody (1:100, Abcam: ab3773).

#### Mechanical Testing Protocols

For mechanical testing, a 2 × 2 mm^2^ piece of cartilage was cored out from the center of each explant using a biopsy punch (Fig. 1b). Explant thickness was measured using vernier callipers then each explant was individually mounted between two impermeable platens on the MTS Bionix 100 (Fig. 1c). Explants were hydrated in phosphate buffered saline (PBS) and an initial tare load of 0.01 N applied. The explants were then subjected to a 20% uniaxial unconfined compressive strain applied at a strain rate of 20%/min. This was immediately followed by a stress relaxation period in which the load was recorded for a further 600 s at a sampling frequency of 10 Hz using a 50 N load cell. Stress–strain and stress–time curves were generated for each specimen and the mechanical properties of the cartilage determined according to the following equations where, *σ*1 and *σ*2 are the stress at 18% and 20% strain during the loading phase and *σ*3 the relaxation stress after 600 s of static compression at 20% strain (Fig. 1d–e).

(1) $$Tangent\;\;\mathrm{mod}ulus(kPa)=\frac{\sigma 2-\sigma 1}{0.02}$$

(2) $$Relaxation\;\;\mathrm{mod}ulus(kPa)=\frac{\sigma 3}{0.2}$$

(3) $$Percentage\;\;\text{relaxation}=\frac{\sigma 2-\sigma 3}{\sigma 2}\times 100$$

The relaxation half‐life (t_1/2_) was also quantified and defined as the time taken for the stress to relax to 50% of the peak stress (*σ*2) measured from the start of the relaxation phase.

### Statistical Analysis

Data represent mean ± standard error of the mean (SEM). Experiments were performed using tissue/cells isolated from at least three donors (N) per experiment. For explant studies, *n* represents a single 5 × 5 mm^2^ explant and for experiments using isolated cells, *n* represents a single well of a tissue culture plate. Statistical analyses were performed using Graph pad (Prism, La Jolla, CA). Following normality testing (Shapiro Wilk test), data was analysed using two‐way ANOVA with post hoc bonferroni *t* tests. In all cases, statistically significant differences relative to the untreated control are indicated at *p* < 0.05 (*), *p* < 0.01 (**), and *p* < 0.001 (***), while difference relative to the IL‐1β treated control are indicated as *p* < 0.05 ($), *p* < 0.01 ($$), and *p* < 0.001 ($$$).

## RESULTS

### Dietary Lithium Does Not Induce Osteoarthritis in Rats Nor Regulate the Expression of Catabolic Enzymes

**Figure 2:**
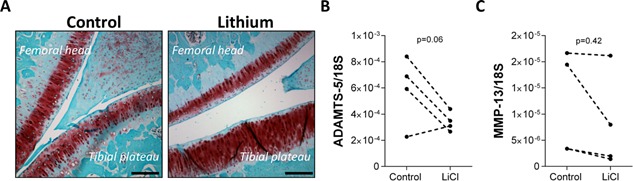
Dietary lithium does not lead to the development of osteoarthritis in vivo. (A) Representative images of cartilage from control rats and rats fed a high lithium diet for 9 months. Cartilage sections were stained with safranin O and fast green. Scale bar represents 200 μm. (B) ADAMTS‐5 and (C) MMP‐13 gene expression in human articular chondrocytes in control and LiCl (50 mM) treated cells. Data is normalized to 18S expression and displayed as an expression ratio for each individual donor (*N* = 4, *n* = 3).

The effects of dietary lithium on cartilage health were examined in vivo using a rat model. Male Wistar rats were fed a diet high in lithium content for 9 months resulting in plasma lithium levels comparable to the therapeutic levels in human plasma (0.8–1.3 mmol/l) and the effects on cartilage health assessed by histological staining (Fig. 2a). In both control and lithium treated groups, the articular cartilage stained positively with safranin O and there were no macroscopic signs of cartilage degradation such as chondrocyte clustering or surface fibrillations.

Furthermore, in human articular chondrocytes 50 mM LiCl did not significantly alter the expression of ADAMTS‐5 (*p* = 0.061, Fig. 2b) or MMP‐13 (*p* = 0.422, Fig. 2c) which are associated with matrix catabolism in OA.

### LiCl Treatment Does Not Influence Chondrocyte Viability, Matrix Catabolism, or Cartilage Mechanics

**Figure 3:**
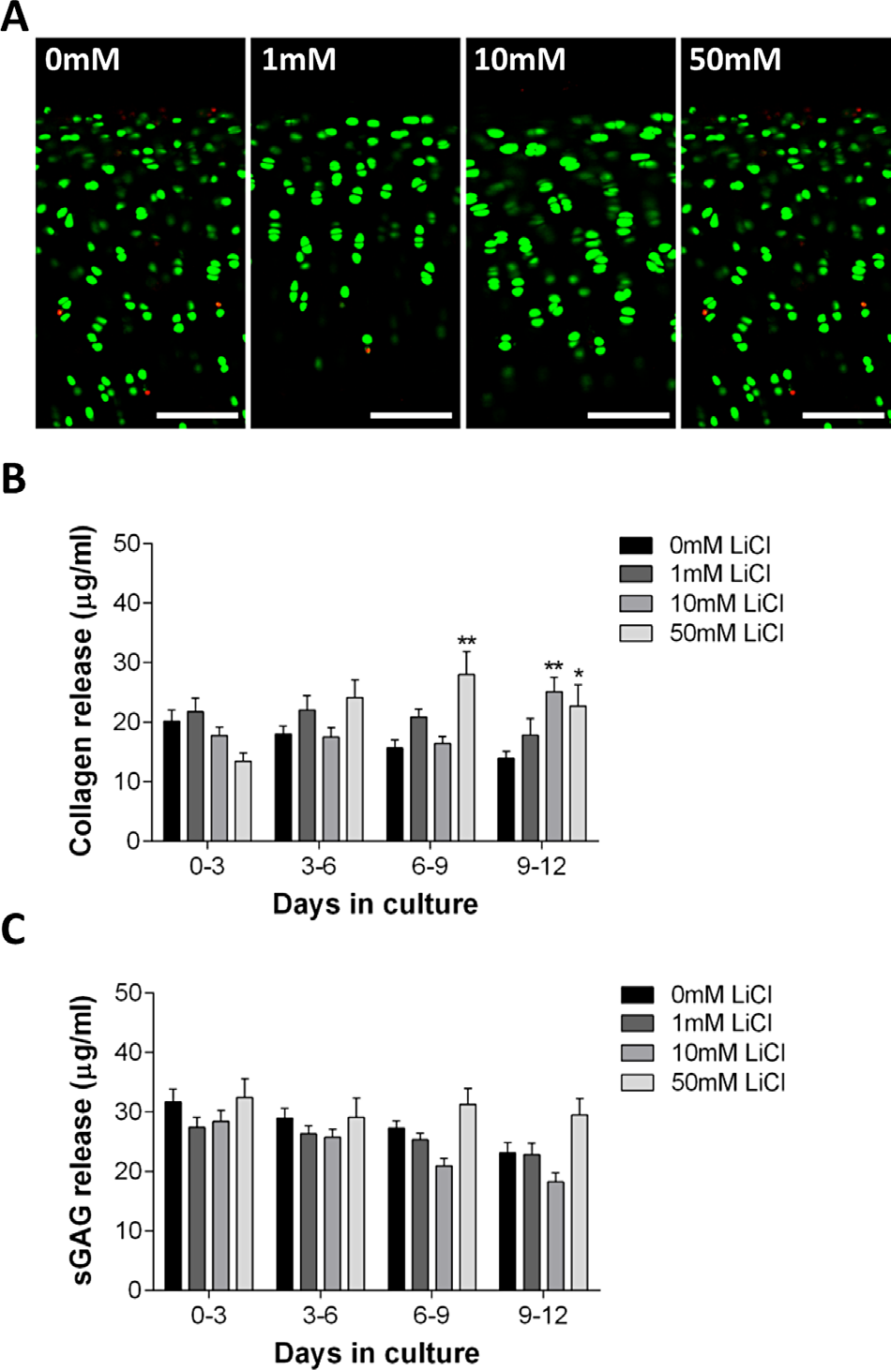
LiCl treatment does not cause cell death or matrix catabolism in vitro. (A) Live/dead staining of car tilage explants treated with 0–50 mM LiCl for 12 days. Live cells (green) were labeled with Calcein AM while dead cells (red) were labeled with Ethidium homodimer‐1. Scale bar = 100 μm. (B) Collagen and (C) sGAG release in response to LiCl treatment over 12 days of culture (*N* = 4, *n* = 8 and *N* = 8, *n* = 16, respectively).

Following 12 days culture in the presence of 0–50 mM LiCl, chondrocyte viability remained above 75% in both control and LiCl treated explants with no statistically significant differences between the two groups (*p* = 0.076, Fig. 3a). Significant release of collagen was observed at 6–9 and 9–12 days at 50 mM LiCl and at 9–12 days for 10 mM LiCl relative to the untreated treatment group; however, total collagen release over the 12‐day treatment period was not significantly altered (10 mM: *p* = 0.720, 50 mM: *p* > 0.999, Fig. 3b). Similarly, the release of sGAG into the culture medium was not significantly increased by LiCl treatment over the 12‐day culture period (Fig. 3c).

The tangent modulus was calculated from the linear portion of the stress–strain curve which was found to be between 18% and 20% strain (Equation (1)). Untreated control explants exhibited a tangent modulus of 2.42 kPa ± 0.36 and a percentage relaxation of 65.97% ± 1.92. These parameters were not significantly altered in the presence of 1, 10, or 50 mM LiCl (Fig. 5). These data indicate that the viability and both the biochemical and mechanical integrity of the cartilage ECM is maintained throughout the course of the experiment indicating that LiCl does not induce cartilage degradation.

### LiCl Inhibits IL‐1β Signaling and Prevents Matrix Catabolism

**Figure 4:**
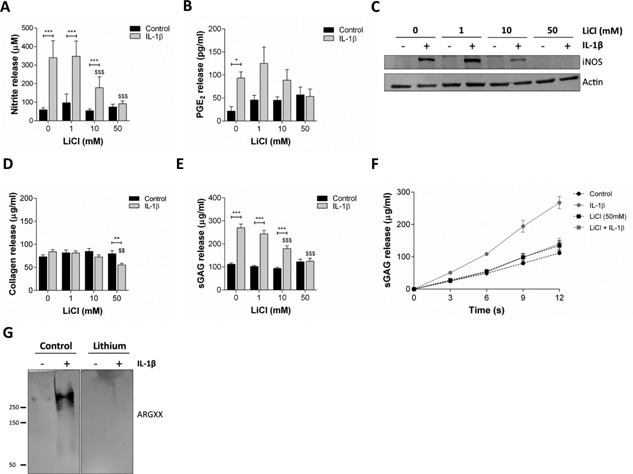
LiCl blocks inflammatory signaling and inhibits matrix catabolism. Cumulative (A) nitrite and (B) PGE_2_ release in response to 0–50 mM LiCl ± 5 ng/ml IL‐1β (*N* = 8, *n* = 16 and *N* = 8, *n* = 8, respectively). (C) Representative western blot for the induction of iNOS expression in isolated primary bovine chondrocytes following IL‐1β treatment for 24 h (*N* = 3). Cumulative (D) collagen and (E) sGAG release in response to 0–50 mM LiCl ± 5 ng/ml IL‐1β (*N* = 4, *n* = 8 and *N* = 8, *n* = 16, respectively). (F) sGAG release over time at 50 mM LiCl (*N* = 8, *n* = 16) (G) Representative western blot for ARGxx aggrecan cleavage epitope.

Previous studies report that LiCl blocks inflammatory signaling and prevents matrix catabolism in cartilage explants.^7^, ^8^ To confirm these results, we assessed the effects of LiCl treatment on the induction of PGE_2_ and NO release in cartilage explants in response to IL‐1β. The concentration of nitrite and PGE_2_ in the culture medium of explants treated with IL‐1β alone was significantly increased by 4.0‐fold (*p* < 0.001, Fig. 4a) and 4.3‐fold (*p* < 0.001, Fig. 4b), respectively after 12 days indicative of release. LiCl inhibited both NO and PGE_2_ release in a dose‐dependent manner such that this response was completely abrogated at 50 mM. Furthermore, we investigated this phenomenon further using isolated cells and found that the IL‐1β dependent induction of iNOS protein was prevented at 50 mM LiCl (Fig. 4c).

IL‐1β treatment alone did not induce significant collagen release (*p* = 0.563, Fig. 4d). However, a significant reduction in basal collagen release was observed upon co‐treatment with IL‐1β and 50 mM LiCl. By contrast, a significant increase in the release of sGAG from explants cultured for 12 days in the presence of IL‐1β indicative of cartilage degradation (*p* < 0.001, Fig. 4e). In the presence of 50 mM LiCl, this response was inhibited such that the IL‐1β treatment group was not significantly different to the control group (*p* > 0.999, Fig. 4e). Analysis of sGAG release over time revealed that IL‐1β induced sGAG release was inhibited from as early as 3 days in culture at 50 mM LiCl (Fig. 4f). Production of the aggrecan ARGxx cleavage product was abolished at 3 days with 50 mM LiCl confirming that the enzymatic degradation of this protein is inhibited (Fig. 4g).

### LiCl Prevents the IL‐1β Induced Loss of Cartilage Mechanical Properties

**Figure 5:**
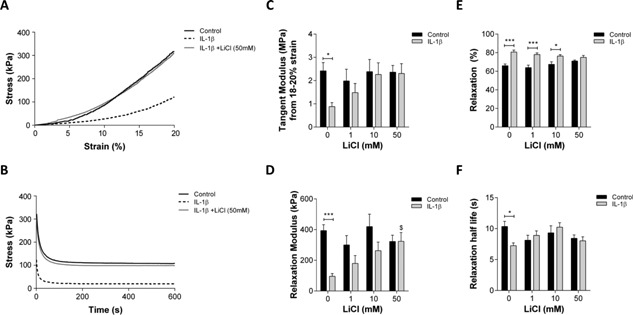
LiCl prevents the IL‐1β induced alterations in cartilage stiffness and stress–relaxation properties. Representative (A) stress–strain and (B) stress–relaxation plots. (C) Tangent modulus (D) relaxation modulus (E) percentage relaxation and (F) relaxation half‐life calculated after 12 days in the presence of 0–50 mM LiCl ± 5 ng/ml IL‐1 β (*N* = 8, *n* = 12).

After 12 days, cartilage explants were subjected to mechanical testing and stress versus strain plots (Fig. 5a) and temporal stress‐relaxation plots (Fig. 5b) generated for each explant. A significant reduction in the tangent modulus was observed for explants treated with IL‐1β alone with values of 2.42 kPa ± 0.36 in control explants and 0.88 kPa ± 0.17 following IL‐1β treatment (*p* = 0.039, Fig. 5c). Similarly, the relaxation modulus was reduced from 393.90 kPa ± 38.7 in control explants to 95.6 kPa ± 18.0 in explants treated with IL‐1β (*p* < 0.001, Fig. 5d). A significant increase in the percentage relaxation was observed from 65.97% ± 1.92 in the control group to 80.68% ± 2.13 in the IL‐1β treated group (*p* < 0.001, Fig. 5e). Moreover, the relaxation half‐life was found to be significantly shorter in the IL‐1β treated group (*p* = 0.019, Fig. 5f) further indicating that the mechanical integrity of the explants are compromised.

Consistent with the effects on matrix catabolism (Fig. 4), LiCl treatment inhibited the loss of mechanical integrity with IL‐1β treatment in a dose‐dependent manner. Thus at 50 mM LiCl the IL‐1β induced reduction in the tangent modulus, equilibrium modulus, and relaxation half‐life and the increase in % stress relaxation were all completely abolished (Fig. 5). For each parameter, there was no statistically significant difference from values measured for untreated controls without IL‐1β (Fig. 5).

## DISCUSSION

The present study is the first to demonstrate that LiCl inhibits the mechanical degradation of articular cartilage induced by the inflammatory cytokine IL‐1β. Moreover, in addition to examining the effects of LiCl on the mechanical properties of cartilage in vitro, it is the first study to investigate the long‐term effects of dietary lithium on cartilage health in vivo.

Frederick et al. report that lithium inhibits a Golgi‐resident PAP 3′‐phosphatase (gPAPP) which indirectly leads to the inhibition of Golgi sulfotransferases resulting in a deficiency in glycosaminoglycan (GAG) sulfation.^10^ Long‐term exposure to lithium might therefore be expected to alter the composition of proteoglycans within the cartilage ECM and negatively affect the mechanical properties of the tissue resulting in cartilage damage. Surprisingly, despite the ability of lithium to inhibit gPAPP with a *k_i_* of <200 μM, we did not observe the development of cartilage lesions or joint abnormalities in vivo (Fig. 2). We would hypothesize that either a functionally redundant enzyme compensates for gPAPP loss, or that the reduction in proteoglycan sulfation is not sufficient to cause cartilage damage in this system.

The compressive properties of articular cartilage are reduced in osteoarthritic tissue relative to healthy tissue. In cartilage explants, the compressive stiffness as determined by a reduction in the dynamic and equilibrium moduli, is also reduced in response to IL‐1β treatment.^18^, ^19^ In the current study, we observed dramatic reductions in both the tangent and relaxation moduli following IL‐1β treatment consistent with a reduction in cartilage stiffness and mechanical degradation of the explant. The compressive stiffness of the articular cartilage is dependent on the high proteoglycan content and the interaction with the collagen fibres.^20^ We did not observe significant collagen release with IL‐1β treatment under the conditions used in the current study (Fig. 4c); therefore, this loss of compressive moduli is likely the result of sGAG release which occurs due to ADAMTS‐mediated cleavage in response to IL‐1β (Fig. 4e–g). Consistent with this observation, stress relaxation tests at constant compressive strain revealed that the percentage stress relaxation of the cartilage explants was significantly increased in the presence of IL‐1β by approximately 15% (Fig. 5e). Relaxation occurs due to the extrusion of the interstitial fluid from the cartilage matrix, thus this observation is also consistent with the loss of negatively charged proteoglycans from the matrix. Remarkably, LiCl treatment blocked inflammatory signaling and produced a dose‐dependent inhibition of mechanical degradation, quantified by the reduction in compressive moduli and increase in stress relaxation. Coupled with the dose‐dependent inhibition of IL‐1β‐induced sGAG release, these data suggest that LiCl prevents the mechanical degradation of cartilage by inhibiting proteoglycan degradation.

One of the many mechanisms by which LiCl influences cellular function is through the inhibition of glycogen synthase kinase β (GSKβ).^21^ GSKβ is a key component of the β‐catenin destruction complex, which acts downstream in the canonical Wnt signaling pathway to promote proteasomal degradation of β‐catenin and inhibition of Wnt target genes. The binding of Wnt ligand to its receptor initiates a signaling cascade which results in GSKβ inhibition and the accumulation of β‐catenin in the nucleus where it promotes TCF/LEF mediated gene transcription. Wnt signaling has been implicated in the development of OA, conditional activation of β‐catenin in articular chondrocyte leads to an osteoarthritic‐like phenotype while increased nuclear localization of β‐catenin has been reported in human OA.^22^ Moreover, a number of Wnt and Wnt‐related proteins such as Wnt16^23^ and Wnt‐induced signaling protein 1 (WISP‐1)^24^ are significantly up regulated in OA tissues. The lack of cartilage damage we observed in vivo is therefore surprising. However, Litherland et al. recently reported that inhibition of GSK alone, using the specific inhibitor CHIR‐99021, does not lead to the development of osteoarthritic lesions in mice consistent with our findings both in rats in vivo and human articular chondrocytes in vitro.^25^ In contrast to the protective effects reported for LiCl, Litherland et al. also observed that specific inhibition of GSK‐enhanced cartilage degradation in response to mechanical injury.^25^ Thus, it is likely that LiCl influences additional cellular targets which counteract its effects on GSK. Indeed, it has recently been shown that lithium inhibits hedgehog signaling in pancreatic ductal adenocarcinoma cells.^26^ Hedgehog signaling is activated in osteoarthritic tissue and inhibiting this pathway in disease models prevents cartilage degradation.^27^ The effects of lithium on chondrocyte hedgehog signaling have not previously been examined; however, we would propose that lithium‐mediated inhibition of hedgehog signaling may also help to maintain the ECM balance and prevent matrix catabolism.

This study suggests that, in addition to the treatment of bipolar disorder, lithium has the potential to reduce matrix catabolism in OA and the associated loss of mechanical functionality that occurs with disease progression. However, it must be emphasized that lithium is a powerful drug the use of which must be carefully monitored in humans to prevent the development of negative side effects. Thus, further studies will be required to determine the safest and most effective method of drug dosing and administration. Regardless, this study adds to a growing body of evidence highlighting the potential of lithium as a novel drug for the treatment of OA.

## AUTHOR'S CONTRIBUTIONS

Acquisition, analysis, and interpretation of data were done by CT, AW, AV, and HY. Substantial contributions to research design, drafting, and critical revision of the manuscript were performed by CT, MK, and CAP. All authors have read and approved the final submitted manuscript.

## References

[1] Alexopoulos LG , Haider MA , Vail TP , et al. 2003 Alterations in the mechanical properties of the human chondrocyte pericellular matrix with osteoarthritis. J Biomech Eng 125:323–333. 12929236 10.1115/1.1579047

[2] Kim E , Guilak F , Haider MA . 2008 The dynamic mechanical environment of the chondrocyte: a biphasic finite element model of cell‐matrix interactions under cyclic compressive loading. J Biomech Eng 130:061009. 19045538 10.1115/1.2978991 PMC2768281

[3] Obeid EM , Adams MA , Newman JH . 1994 Mechanical properties of articular cartilage in knees with unicompartmental osteoarthritis. J Bone Joint Surg Br 76:315–319. 8113301

[4] Boschetti F , Peretti GM . 2008 Tensile and compressive properties of healthy and osteoarthritic human articular cartilage. Biorheology 45:337–344. 18836234

[5] Arthritis Research UK 2013. Osteoarthritis in general practice: Data and perspectives.

[6] Kapoor M , Martel‐Pelletier J , Lajeunesse D , et al. 2011 Role of proinflammatory cytokines in the pathophysiology of osteoarthritis. Nat Rev Rheumatol 7:33–42. 21119608 10.1038/nrrheum.2010.196

[7] Hui W , Litherland GJ , Jefferson M , et al. 2010 Lithium protects cartilage from cytokine‐mediated degradation by reducing collagen‐degrading MMP production via inhibition of the P38 mitogen‐activated protein kinase pathway. Rheumatology (Oxford) 49:2043–2053. 20634235 10.1093/rheumatology/keq217

[8] Minashima T , Zhang Y , Lee Y , et al. 2014 Lithium protects against cartilage degradation in osteoarthritis. Arthritis Rheumatol 66:1288–1236. 10.1002/art.38373 24470226

[9] Zhang WV , Jullig M , Connolly AR , et al. 2005 Early gene response in lithium chloride induced apoptosis. Apoptosis 10:75–90. 15711924 10.1007/s10495-005-6063-x

[10] Frederick JP , Tafari AT , Wu SM , et al. 2008 A role for a lithium‐inhibited Golgi nucleotidase in skeletal development and sulfation. Proc Natl Acad Sci USA 105:11605–11612. 18695242 10.1073/pnas.0801182105 PMC2575314

[11] Chowdhury TT , Bader DL , Lee DA . 2001 Dynamic compression inhibits the synthesis of nitric oxide and PGE(2) by IL‐1beta‐stimulated chondrocytes cultured in agarose constructs. Biochem Biophys Res Commun 285:1168–1174. 11478777 10.1006/bbrc.2001.5311

[12] Larionov A , Krause A , Miller W . 2005 A standard curve based method for relative real time PCR data processing. BMC Bioinform 6:62. 10.1186/1471-2105-6-62 PMC1274258 15780134

[13] Farndale RW , Sayers CA , Barrett AJ . 1982 A direct spectrophotometric microassay for sulfated glycosaminoglycans in cartilage cultures. Connect Tissue Res 9:247–248. 6215207 10.3109/03008208209160269

[14] Kafienah W , Sims TJ . 2004 Biochemical methods for the analysis of tissue‐engineered cartilage. Methods Mol Biol 238:217–230. 14970450 10.1385/1-59259-428-x:217

[15] Lee DA , Frean SP , Lees P , et al. 1998 Dynamic mechanical compression influences nitric oxide production by articular chondrocytes seeded in agarose. Biochem Biophys Res Commun 251:580–585. 9792816 10.1006/bbrc.1998.9520

[16] Wann AK , Chapple JP , Knight MM . 2014 The primary cilium influences interleukin‐1beta‐induced NFkappaB signalling by regulating IKK activity. Cell Signal 26:1735–1742. 24726893 10.1016/j.cellsig.2014.04.004 PMC4064300

[17] Thoms BL , Dudek KA , Lafont JE , et al. 2013 Hypoxia promotes the production and inhibits the destruction of human articular cartilage. Arthritis Rheum 65:1302–1312. 23334958 10.1002/art.37867

[18] Palmer AW , Wilson CG , Baum EJ , et al. 2009 Composition‐function relationships during IL‐1‐induced cartilage degradation and recovery. Osteoarthritis Cartilage 17:1029–1039. 19281879 10.1016/j.joca.2009.02.009 PMC2745941

[19] Bonassar LJ , Sandy JD , Lark MW , et al. 1997 Inhibition of cartilage degradation and changes in physical properties induced by IL‐1beta and retinoic acid using matrix metalloproteinase inhibitors. Arch Biochem Biophys 344:404–412. 9264555 10.1006/abbi.1997.0205

[20] Bader DL , Kempson GE . 1994 The short‐term compressive properties of adult human articular cartilage. Biomed Mater Eng. 4:245–256. 7950872

[21] Klein PS , Melton DA . 1996 A molecular mechanism for the effect of lithium on development. Proc Natl Acad Sci USA 93:8455–8459. 8710892 10.1073/pnas.93.16.8455 PMC38692

[22] Zhu M , Tang D , Wu Q , et al. 2009 Activation of beta‐catenin signaling in articular chondrocytes leads to osteoarthritis‐like phenotype in adult beta‐catenin conditional activation mice. J Bone Miner Res 24:12–21. 18767925 10.1359/JBMR.080901 PMC2640321

[23] Dell'accio F , De Bari C , Eltawil NM , et al. 2008 Identification of the molecular response of articular cartilage to injury, by microarray screening: Wnt‐16 expression and signaling after injury and in osteoarthritis. Arthritis Rheum 58:1410–1421. 18438861 10.1002/art.23444

[24] Blom AB , Brockbank SM , van Lent PL , et al. 2009 Involvement of the Wnt signaling pathway in experimental and human osteoarthritis: Prominent role of Wnt‐induced signaling protein 1. Arthritis Rheum 60:501–512. 19180479 10.1002/art.24247

[25] Litherland GJ , Hui W , Elias MS , et al. 2014 Glycogen synthase kinase 3 inhibition stimulates human cartilage destruction and exacerbates murine osteoarthritis. Arthritis Rheumatol 66:2175–2187. 24757033 10.1002/art.38681

[26] Peng Z , Ji Z , Mei F , et al. 2013 Lithium inhibits tumorigenic potential of PDA cells through targeting hedgehog‐GLI signaling pathway. PLoS ONE 8:e61457. 23626687 10.1371/journal.pone.0061457 PMC3634073

[27] Lin AC , Seeto BL , Bartoszko JM , et al. 2009 Modulating hedgehog signaling can attenuate the severity of osteoarthritis. Nat Med 15:1421–1425. 19915594 10.1038/nm.2055

